# Study on the mechanism of hyperoside in affecting the biological progression and radiosensitivity of esophageal carcinoma by modulating the STAT3/AKT/ERK pathway

**DOI:** 10.17305/bb.2024.11201

**Published:** 2024-11-16

**Authors:** Hongmei Yin, Zhongxia Yuan, Xiumei Han, Die Jiang, Duojie Li, FengLi Song

**Affiliations:** 1 Department of Radiotherapy, First Affiliated Hospital of Bengbu Medical University, Longzihu District, Bengbu, Anhui Province, China; 2 Department of Oncology, Beijing University of Chinese Medicine, Beijing, China; 3 Department of Oncology, Beijing University of Chinese Medicine Third Affiliated Hospital, Andingmenwai, Chaoyang District, Beijing, China

**Keywords:** Hyperoside, HYP, esophageal carcinoma, EC, STAT3/AKT//ERK pathway, radiosensitivity

## Abstract

Hyperoside (HYP) exhibits diverse pharmacological effects and holds potential for enhancing chemotherapy sensitivity. However, few studies have reported the impact of HYP on the malignant progression of esophageal carcinoma (EC) and its sensitivity to radiotherapy. The impact of HYP on the viability of EC cells (TE-1 and KYSE-150) was assessed using Cell Counting Kit-8 (CCK-8) assays. The biological characteristics and radiosensitivity of EC cells following HYP treatment were evaluated through clone formation experiments, flow cytometry, scratch wound-healing assays, and transwell migration and invasion assays. Western blot analysis was performed to determine the levels of proteins associated with cell death and epithelial-mesenchymal transition (EMT), as well as to explore whether HYP interferes with the radiosensitivity of EC cells via the STAT3/AKT/ERK pathways. Finally, a subcutaneous graft tumor model was constructed to investigate the effects of HYP and X-ray treatments on *in vivo* tumor growth. The findings indicated a dose-dependent decrease in the survival rate of KYSE-150 and TE-1 cells following HYP treatment. HYP treatment also inhibited cell proliferation, invasion, migration, and EMT, while increasing the apoptotic rate and radiosensitivity of the cells. Notably, HYP suppressed the malignant progression of EC and enhanced radiosensitivity via the STAT3/AKT/ERK pathway. Moreover, HYP impaired the growth of EC tumors in mice, with the combined HYP and X-ray treatment exerting a stronger inhibitory effect. In conclusion, HYP increases the radiosensitivity of esophageal carcinoma cells, offering considerable promise for application in the clinical treatment of EC.

## Introduction

Esophageal carcinoma (EC) ranks as the sixth leading cause of cancer-related deaths globally and is the eighth most frequently diagnosed cancer type [1]. Histologically, approximately 90% of EC cases are esophageal squamous cell carcinoma (ESCC) [2, 3]. EC is characterized by its high incidence, elevated mortality rates, and poor prognosis, with the majority of patients presenting symptoms in advanced stages (II–IV) [1, 4]. In 2020, there were an estimated 604,100 newly diagnosed EC cases and 544,100 deaths attributed to the disease worldwide. By 2040, these figures are projected to rise to 957,000 new cases and 880,000 deaths [5]. Radiotherapy (RT) remains a cornerstone of EC treatment [6]. However, despite significant advancements in RT equipment and techniques, local recurrence due to radioresistance remains common, with a 5-year survival rate of less than 30% [7, 8]. Therefore, enhancing the radiosensitivity of EC to improve treatment outcomes has become a key focus of current research. Hyperoside (HYP), a flavonol glycoside found in numerous botanical sources (e.g., Hypericum perforatum, Forsythia, Dodder, and Hawthorn flowers), is known for its vascular protective, digestive system regulatory, anti-oxidative, and anti-aging properties [9, 10]. In recent years, HYP’s anti-tumor properties have garnered significant attention. Studies have shown that HYP effectively inhibits the progression of various malignancies, including skin cancer [11], lung cancer [12], pancreatic cancer [13], and non-small cell lung cancer [14]. For instance, Sun et al. [15] demonstrated that HYP reverses paclitaxel resistance in breast cancer cells and enhances chemotherapy sensitivity by blocking the TLR4 signaling pathway. Similarly, Zhu et al. [16] found that HYP increases the sensitivity of cisplatin-resistant ovarian cancer cells to cisplatin treatment. However, there is currently no research investigating whether HYP can enhance the radiosensitivity of EC. AKT serine–threonine kinase (AKT), extracellular signal-regulated kinase (ERK), and signal transducer and activator of transcription 3 (STAT3) are critical pathways involved in cancer cell proliferation and metastasis, playing essential roles in EC progression [17–19]. For example, Xu et al. [19] reported that NETO2 activates the ERK and AKT signaling pathways, promoting the growth and metastasis of EC cells. Additionally, Hu et al. [20] showed that inhibiting STAT3 signaling suppresses EC progression both *in vitro* and *in vivo*. Notably, HYP has been found to inhibit liver cancer cell growth by targeting the PI3K/AKT signaling pathway [21]. Meanwhile, TG101209, a JAK2 inhibitor that blocks STAT3 activation, has been shown to enhance lung cancer cell radiosensitivity by reducing survivin expression, with downstream effects on PI3K/AKT and Ras/MAPK/ERK pathways [22].

Based on these findings, we hypothesized that HYP could inhibit EC progression and improve its radiosensitivity by targeting the STAT3/AKT/ERK pathway. To test this hypothesis, this study investigated the effects of HYP on EC cell malignancy and radiosensitivity *in vitro* and explored whether its actions are mediated through the STAT3/AKT/ERK pathway. KYSE-150 and TE-1 cell lines, which are widely used to study EC radiosensitivity [23, 24], were selected for the experiments. Additionally, an *in vivo* EC tumor model was developed using subcutaneous xenografts in mice to examine the combined effects of HYP and RT on tumor progression. This study aims to clarify the role of HYP in regulating EC radiosensitivity and its underlying mechanisms, providing new insights for the clinical treatment of this malignancy.

## Materials and methods

### Cell cultivation

EC cell lines KYSE-150 (SNL-344, STR verified) and TE-1 (SNL-209, STR verified) were sourced from Sunncell Biotechnology Co., Ltd. (Wuhan, Hubei, China). These cells were cultured in RPMI-1640 medium (Gibco, Grand Island, NY, USA) supplemented with 1% streptomycin–penicillin solution (Gibco) and 10% fetal bovine serum (Sigma-Aldrich, St. Louis, MO, USA). The cultures were maintained in a humidified incubator with 5% CO_2_ at 37 ^∘^C. Passaging was carried out every three days, and the culture medium was replaced every other day.

### Cell Counting Kit-8 (CCK-8) assay

Following the method described by Yang et al. [25], 5000 TE-1 and KYSE-150 cells were seeded into each well of a 96-well culture plate. After the cells adhered to the walls, the original medium was removed, and the cells were incubated with media containing different concentrations of HYP (0, 12.5, 25, 50, or 100 µg/mL) (HY-N0452, MedChemExpress, USA) [15]. HYP was dissolved in 10% DMSO and diluted to the appropriate concentrations using saline. The final DMSO concentration was below 0.05%, a level confirmed to be non-toxic to the cells in a preliminary experiment. After 48 h of culture, 100 µL of culture medium containing 10% CCK-8 assay solution (C0038, Beyotime, China) was added to each well, and the plate was incubated for an additional 2 h at 37 ^∘^C. The optical density at 450 nm (OD450) was then measured using a microplate reader (1410101, Thermo Fisher Scientific, USA).

### Clone formation experiment

TE-1 and KYSE-150 cells were trypsinized, resuspended, and counted. Five hundred cells were seeded into each well of a 6-well cell culture plate and incubated in HYP-containing medium for 48 h. The medium was then discarded and replaced with fresh, drug-free medium. This fresh medium was changed every 2–3 days for 14 days under standard culture conditions (37 ^∘^C, 5% CO_2_).

Once visible cell clusters formed, the wells were washed twice with 1× PBS. Next, the cells were fixed with 4% paraformaldehyde (P1110, Solarbio, Beijing, China) for 20 min. After discarding the fixative, the cells were stained with 0.1% crystal violet (C0775, Sigma-Aldrich) for 15 min. Finally, images of the clones were captured and counted using an inverted fluorescent microscope (MF52-N, Guangzhou Ming-Mei Technology Co., Ltd, Guangdong, China).

### Flow cytometry (FCM) assay

Log-phase cells (TE-1 and KYSE-150) were harvested and centrifuged at 3000× *g* for 8 min. The cells were then washed twice with 1× PBS solution and resuspended in 500 µL of binding solution. Next, 5 µL of Annexin-V-FITC (HY-K1073, MedChemExpress) and 5 µL of propidium iodide (ST1569, Beyotime) were added to the cell suspension. The mixture was gently stirred and incubated for 15 min at room temperature in the dark. After incubation, the samples were transferred into specialized tubes and analyzed using a flow cytometer (BD FACSCalibur™, BD Biosciences, San Jose, CA, USA) to evaluate cell apoptosis.

### Western blot assay

Protein extraction from cells and tissues was performed using RIPA lysis buffer (P0013, Beyotime). Protein concentrations were quantified using a BCA protein assay kit (P0012, Beyotime). The extracted proteins were separated by SDS-PAGE using a 10% polyacrylamide gel and subsequently transferred onto a PVDF membrane (Invitrogen, Carlsbad, CA, USA). The membrane was blocked with 5% non-fat milk for 3 h and incubated overnight at 4 ^∘^C with primary antibodies, including:

Anti-Cleaved-Caspase3 (ab2302, 1:500, Abcam), Anti-Cleaved-Caspase9 (PA5-17913, 1:1000, Invitrogen), Anti-Bcl-2 (ab59348, 1:500, Abcam), Anti-Vimentin (PA5-27231, 1:10,000, Invitrogen), Anti-E-cadherin (ab314063, 1:100, Abcam), Anti-N-cadherin (ab18203, 1:1000, Abcam), Anti-p-STAT3 (710093, 1:100, Invitrogen), Anti-STAT3 (710077, 1:50, Invitrogen), Anti-p-AKT (44-621G, 1:1000, Invitrogen), Anti-AKT (44-609G, 1:1000, Invitrogen), Anti-p-ERK (44-680G, 1:1000, Invitrogen), and Anti-ERK (61–7400, 1:2000, Invitrogen). On the following day, the membrane was washed three times and incubated with a goat anti-rabbit IgG secondary antibody (31460, 1:10,000, Invitrogen) for 2 h. Image development was performed using chemiluminescence, and the protein bands were visualized using a gel imaging system (WD-9413B, Beijing Liuyi Biotechnology Co., Ltd., Beijing, China). The grayscale values of the bands were analyzed using ImageJ software. The relative protein expression levels were calculated as the ratio of each target protein’s grayscale value to that of GAPDH (PA1-987, 1:1000, Invitrogen). Edits focused on smoothing awkward phrasing, ensuring consistent formatting, and improving clarity without altering technical content.

### Scratch assays

Log-phase TE-1 and KYSE-150 cells were seeded into 6-well plates pre-marked with horizontal lines. Once the cells reached confluency at the bottom of the wells, a vertical scratch was gently created across the pre-marked lines using a 20 µL sterile pipette tip. The cells were then rinsed twice with PBS to remove debris, followed by the addition of a serum-free RPMI-1640 medium. The plates were incubated at 37 ^∘^C, and the progression of scratch healing was observed at 0 and 24 h. Statistical analysis of the wound area reduction was performed using ImageJ software.

### Transwell assays

Matrigel (Sigma-Aldrich) was first diluted in RPMI-1640 medium without serum. Then, 100 µL of the diluted gel was added to each transwell (Corning, Tewksbury, MA, USA) and incubated overnight. The following day, the remaining liquid in the transwells was carefully removed, and RPMI-1640 medium without serum was added to hydrate the basement membrane. Next, 200 µL of a TE-1 or KYSE-150 cell suspension was seeded into each transwell, while 800 µL of RPMI-1640 medium containing 10% fetal bovine serum was added to the lower chamber. After 36 h of incubation, cotton swabs were used to remove the Matrigel and any non-invading cells from the membrane. To fix the invaded cells, 4% paraformaldehyde was added to the lower compartments and incubated for 30 min. The fixed cells were then stained with a 0.1% crystal violet solution for 5 min and rinsed twice with 1× PBS. Finally, each transwell was imaged from random fields using an inverted fluorescent microscope under high magnification to quantify the invaded cells.

### Hypodermic tumor formation in nude mice

Following the methods described by Liu et al. [26, 27], healthy adult BALB/c nude mice were obtained from Vitalriver (Beijing, China) and housed under controlled environmental conditions: a constant temperature of 22 ^∘^C and a humidity range of 55%–60%. The lighting schedule alternated between a 12-h light phase and a 12-h dark phase. The mice were randomly divided into four groups, with six mice in each group. Each mouse was injected subcutaneously with 0.2 mL of KYSE-150 cell suspension in the logarithmic growth phase (1.5 × 10^ImEquation2^ cells per mouse). According to the method of Feng et al. [28], mice in the IR+HYP group and the HYP group were administered HYP orally at a dose of 6.0 mg/kg every two days for 14 consecutive days. Following a previous study [29], mice in the IR group and the IR+HYP group were subjected to 4 Gy 6MV-X-ray irradiation at the tumor site on the 15th, 20th, and 25th days after cell injection. The control group received no treatment. On the 28th day, the mice were euthanized under anesthesia. Tumors were excised, and their sizes were measured using calipers. Tumor masses were weighed, and photographs of the tumors were taken. The animal experiment protocol was approved by the Experimental Animal Ethics Committee at the Third Affiliated Hospital of Beijing University of Chinese Medicine.

### Immunohistochemistry

After fixation with a 4% paraformaldehyde solution, tumor samples were dehydrated, embedded in paraffin, and sectioned into 4–5 µm thick slices using standard procedures. The sections were then deparaffinized in xylene (16446, Sigma-Aldrich) for 5 min. Antigen retrieval was performed through microwave treatment. Subsequently, the sections were incubated with the primary antibody, anti-Ki-67 (MA5-14520, 1:100, Invitrogen), at 37 ^∘^C for 90 min, followed by treatment with a goat anti-rabbit IgG secondary antibody for 1 h. Visualization was achieved using a DAB solution (DA1010, Solarbio), and the reaction was stopped by rinsing the slices with distilled water. Finally, the sections were counterstained with Mayer’s hematoxylin (MHS16, Sigma-Aldrich) and sealed with neutral gum.

### TUNEL staining

The extent of cell apoptosis was assessed using a TUNEL assay kit (C1091, Beyotime) to detect apoptotic cells. Mouse tumor tissues were first fixed in 4% paraformaldehyde, followed by paraffin embedding, sectioning, and deparaffinization using xylene. The tissue sections were then hydrated through graded ethanol solutions (100%, 90%, and 70%) for 5 min each. After hydration, the sections were treated with Proteinase K working solution (20 µg/mL, ST532, Beyotime) without DNase at 37 ^∘^C for 30 min. Following three washes with 1× PBS, the TUNEL detection solution was applied, and the sections were incubated in the dark for 1.5 h. DAPI staining solution was then added, and the sections were incubated in darkness for an additional 10 min. Finally, the samples were observed and imaged using a microscope (XK-DZ004, SINICO Optical Instrument Co., Ltd., Shenzhen, China).

### Ethical statement

This study was approved by the Beijing University of Chinese Medicine Third Affiliated Hospital (Approval No. 20221277).

### Statistical analysis

All assays and experiments were conducted with at least three repetitions, and the results are expressed as the mean ± standard deviation. Figures were created using Prism software (GraphPad Prism 9.0). Statistical analyses were performed using SPSS 26.0 software (IBM SPSS Statistics 26). Prior to comparisons, normality and homogeneity of variance tests were conducted to ensure the data followed a normal distribution and had homogeneous variance. For group comparisons, Student’s *t*-test and one-way analysis of variance (ANOVA) were applied. A *P* value of <5 was considered statistically significant.

## Results

### HYP inhibits malignant biological properties of EC cells

**Figure 1.**
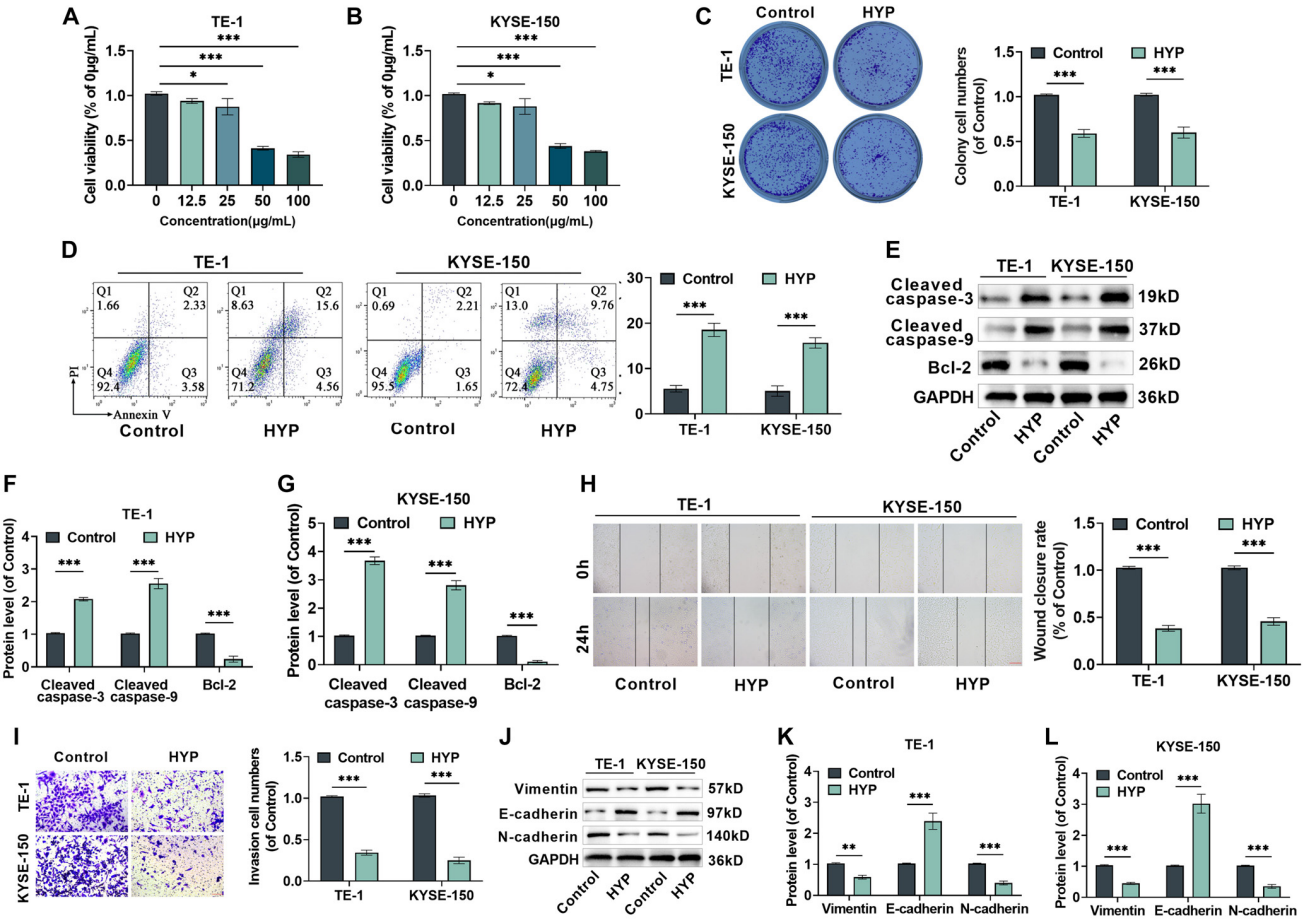
**Variations in malignant biological properties of EC cells following HYP treatment.** (A--B) Evaluating the impacts of a 48-h exposure to HYP at different concentrations on the survival rate of endothelial cells. After treatment with 50 µg/mL of HYP, the viability of EC cells was reduced to below 50%. Hence, 50 µg/mL of HYP was used in subsequent experiments. (C) Clone formation experiments were executed to examine the influence of HYP administration on cellular cloning capacity. (D) Detecting the apoptosis rate of cells treated with HYP. (E–G) The expression levels of Cleaved-Caspase3, Cleaved-Caspase9, and Bcl-2 proteins. (H) Wound healing assay determined migration after HYP treatment, with the wound closure rate subsequently computed, 10×, bar ═ 200 µm. (I) Transwell assay was used to evaluate the invasion of EC cells after HYP treatment and quantitatively analyze the cell invasion rate, 40×, bar ═ 50 µm. (J–L) Assessing the levels of Vimentin, E-cadherin, and N-cadherin proteins. *n* ═ 3. *P < 0.05 and ***P < 0.001 vs control. HYP: Hyperoside; EC: Esophageal cancer.

The outcomes of the CCK-8 assays evaluating EC cell viability after HYP treatment revealed a dose-dependent reduction in cell viability for TE-1 and KYSE-150 cells when exposed to 25, 50, and 100 µg/mL of HYP (Figure 1A and 1B). Specifically, treatment with 50 µg/mL HYP reduced cell survival rates to below 50%. Consequently, this concentration of HYP (50 µg/mL) was selected for subsequent assays and experiments. Clone formation assays further demonstrated a significant decrease in the ability of TE-1 and KYSE-150 cells to form colonies in the presence of HYP (Figure 1C).

HYP’s influence on cell apoptosis was then assessed using FCM and western blot methods. Apoptosis rates in TE-1 and EC9706 cells increased significantly following HYP treatment (Figure 1D). This was accompanied by a marked elevation in the levels of apoptosis-related proteins Cleaved-Caspase3 and Cleaved-Caspase9, as well as a substantial reduction in Bcl-2 protein levels (Figure 1E–1G).

Additionally, scratch and transwell assays confirmed that HYP significantly impaired the migration and invasion abilities of TE-1 and KYSE-150 cells, as shown in Figure 1H and 1I. Furthermore, HYP treatment led to a notable increase in E-cadherin protein levels and significant reductions in Vimentin and N-cadherin protein levels (Figure 1J–1L). These findings indicate that HYP inhibits the epithelial–mesenchymal transition (EMT) of TE-1 and KYSE-150 cells.

In conclusion, these results demonstrate that HYP suppresses the proliferation, migration, invasion, and EMT of EC cells while inducing apoptosis.

### HYP enhances radiosensitivity of EC cells

**Figure 2.**
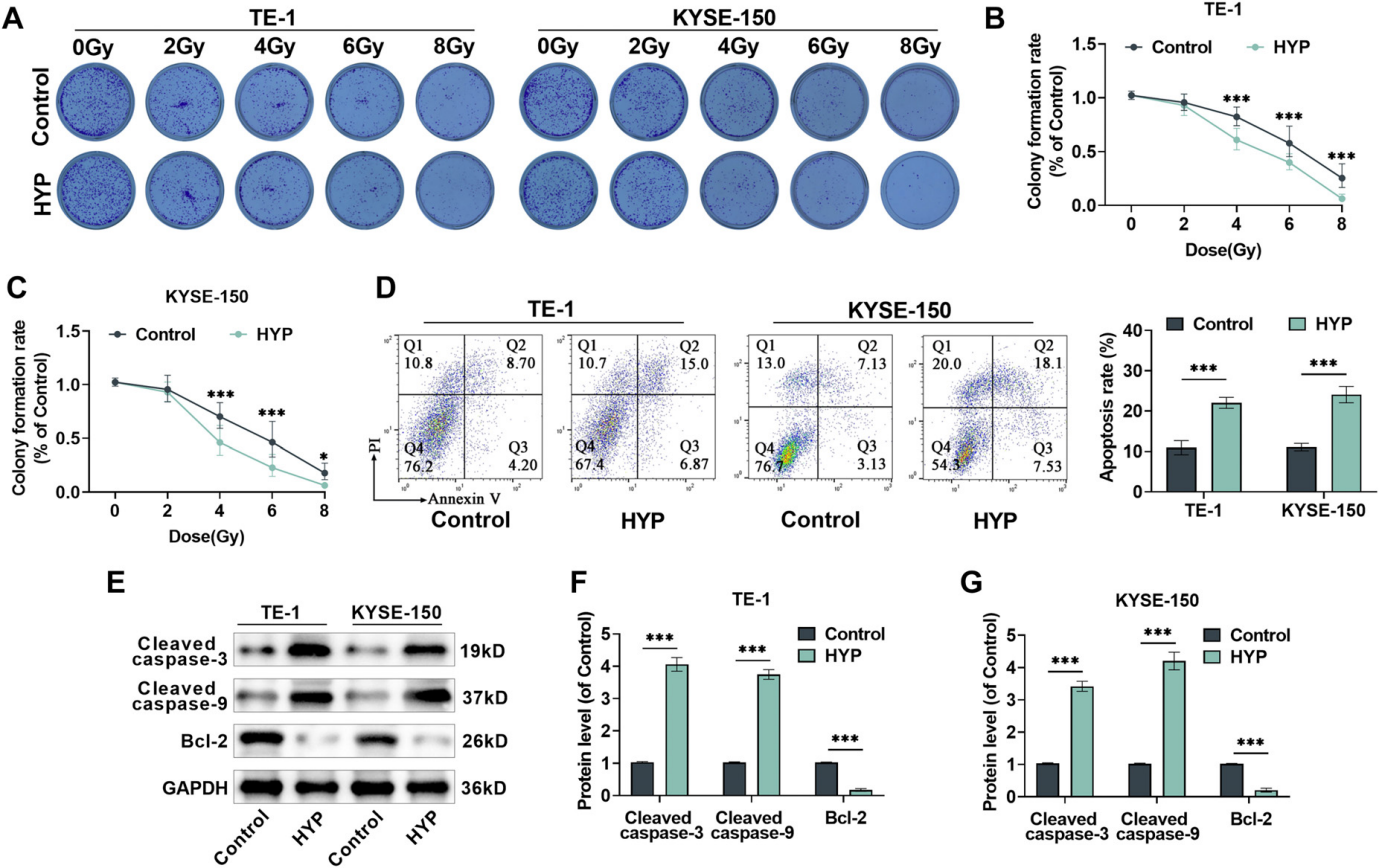
**Variations in radiosensitivity of EC cells following HYP treatment.** (A) Following different doses of X-ray irradiation on TE-1 and KYSE-150 cells for two weeks, clone formation assays were to assess the impact of HYP treatment on the cells’ clonality. (B and C) Quantitative analysis was made of the cells’ cloning efficiency after HYP treatment. After exposure to 4 Gy of X-ray radiation, the cloning rates of TE-1 and KYSE-150 cells began to decrease significantly. Subsequent experiments adopted an X-ray radiation dose of 4 Gy. (D) Examining the effect of HYP treatment post-radiation on cell apoptosis rate. (E–G) Measuring the expression levels of Cleaved-Caspase3, Cleaved-Caspase9, and Bcl-2 proteins. *n* ═ 3. *P < 0.05, ***P < 0.001 vs control. (The control group was treated by X-ray radiation only; the HYP group was treated with HYP post-X-ray radiation.) HYP: Hyperoside; EC: Esophageal cancer.

Subsequently, the impact of HYP on the radiosensitivity of EC cells was assessed. After two weeks of irradiation with 6 MV X-rays at varying doses (2, 4, 6, and 8 Gy), a clonogenic assay demonstrated a significant reduction in the colony-forming efficiency of TE-1 and KYSE-150 cells at a radiation dose of 4 Gy, with a dose-dependent effect observed (Figure 2A–2C). Notably, HYP treatment further diminished colony-forming efficiency, confirming that HYP enhances the effectiveness of X-ray irradiation. Based on these findings, subsequent experiments were conducted using a radiation dose of 4 Gy.

FCM and western blot analyses revealed that, compared to X-ray treatment alone (control group), HYP treatment significantly increased the apoptotic rate in TE-1 and KYSE-150 cells (Figure 2D). Additionally, levels of Cleaved-Caspase3 and Cleaved-Caspase9 were markedly elevated (Figure 2E–2G), while Bcl-2 expression was suppressed. These changes suggest that HYP promotes apoptosis in EC cells following RT and enhances their sensitivity to X-rays under the same conditions.

### HYP modulates the STAT3/AKT/ERK pathway

**Figure 3.**
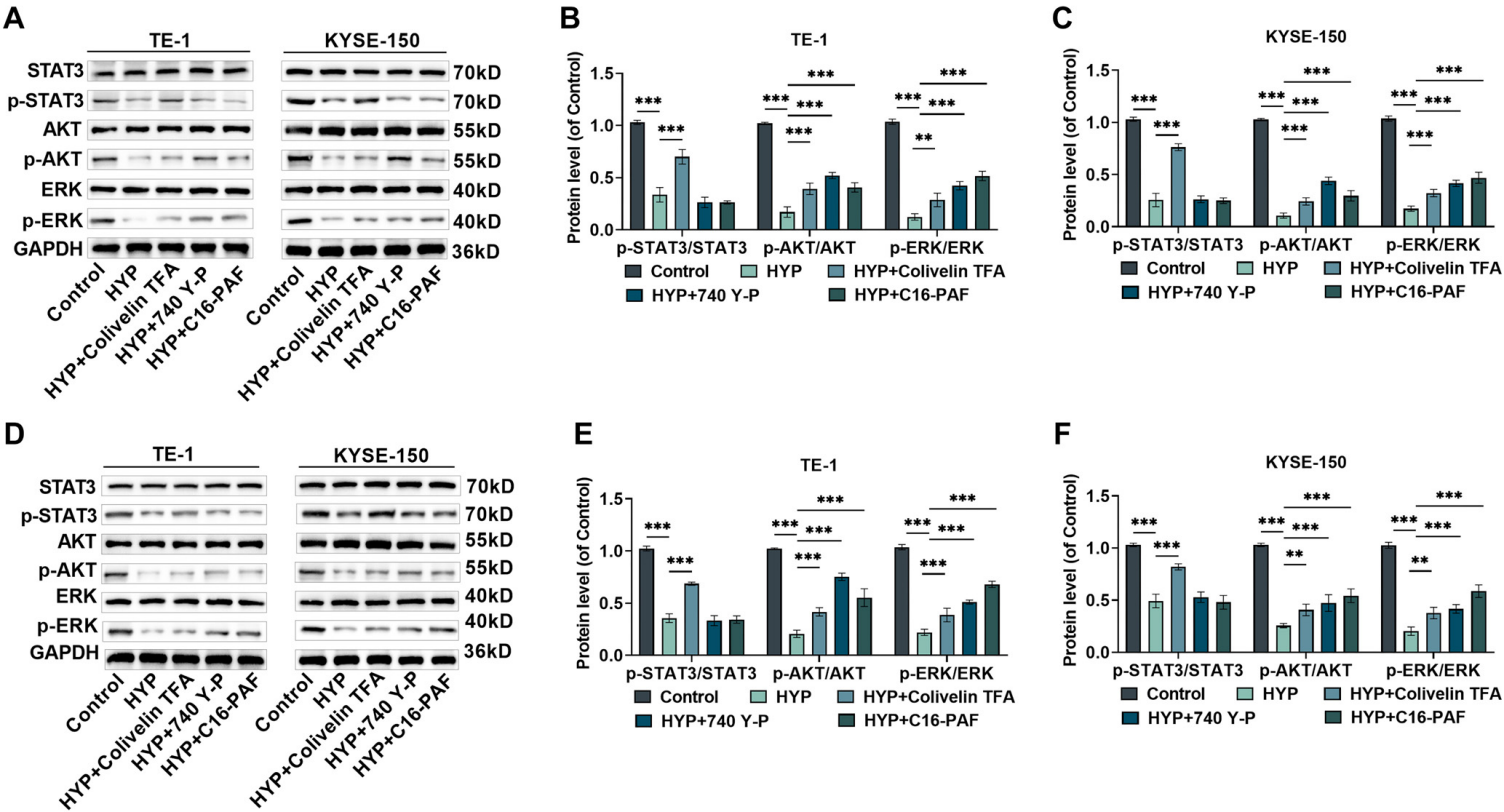
**The influence of HYP on the STAT3/AKT/ERK pathway.** (A) Western blot bands of p-STAT3, STAT3, p-AKT, AKT, p-ERK, and ERK in TE-1 and KYSE-150 cells after being treated with HYP or STAT3/AKT/ERK signaling pathway agonists; (B and C) Quantitative analysis of p-STAT3/STAT3, p-AKT/AKT, and p-ERK/ERK levels in the cells; (D) Western blot bands of p-STAT3, STAT3, p-AKT, AKT, p-ERK, and ERK in TE-1 and KYSE-150 cells following treatment with HYP or STAT3/AKT/ERK signaling pathway agonists after being exposed to 6 MV X-ray radiation (4 Gy); (E and F) Quantitative analysis of p-STAT3/STAT3, p-AKT/AKT, and p-ERK/ERK levels in different treatment groups post-X-ray radiation. *n* ═ 3. ***P < 0.001 vs corresponding control. (Each group in [A–C] is untreated for X-ray radiation, while each group in [D–F] is treated for X-ray radiation). HYP: Hyperoside; EC: Esophageal cancer; ERK: Extracellular signal-regulated kinase; STAT3: Signal transducer and activator of transcription 3; AKT: AKT serine–threonine kinase.

Further investigation examined the phosphorylation levels of STAT3, AKT, and ERK proteins to elucidate the mechanisms by which HYP suppresses the malignant progression of EC and enhances its radiosensitivity. Western blot analysis revealed that HYP significantly reduced the phosphorylation levels of STAT3, AKT, and ERK in the TE-1 and KYSE-150 cell lines (Figure 3A–3C). Interestingly, upon the addition of the STAT3 agonist Colivelin TFA, the phosphorylation levels of STAT3, AKT, and ERK increased. Similarly, when the AKT agonist 740 Y-P or the ERK agonist C16-PAF was added, the levels of p-AKT and p-ERK increased, while p-STAT3 levels remained unchanged. This suggests that STAT3 activation occurs upstream of ERK and AKT activation. These findings indicate that HYP modulates the STAT3/AKT/ERK signaling pathway.

Moreover, in comparison to X-ray treatment alone, the combination of X-ray and HYP treatment further attenuated the phosphorylation of ERK, STAT3, and AKT proteins in TE-1 and KYSE-150 cells (Figure 3D–3F). These results confirm that HYP effectively modulates the STAT3/AKT/ERK signaling pathway.

### HYP inhibits progression of EC via the modulation of STAT3/AKT/ERK pathway

**Figure 4.**
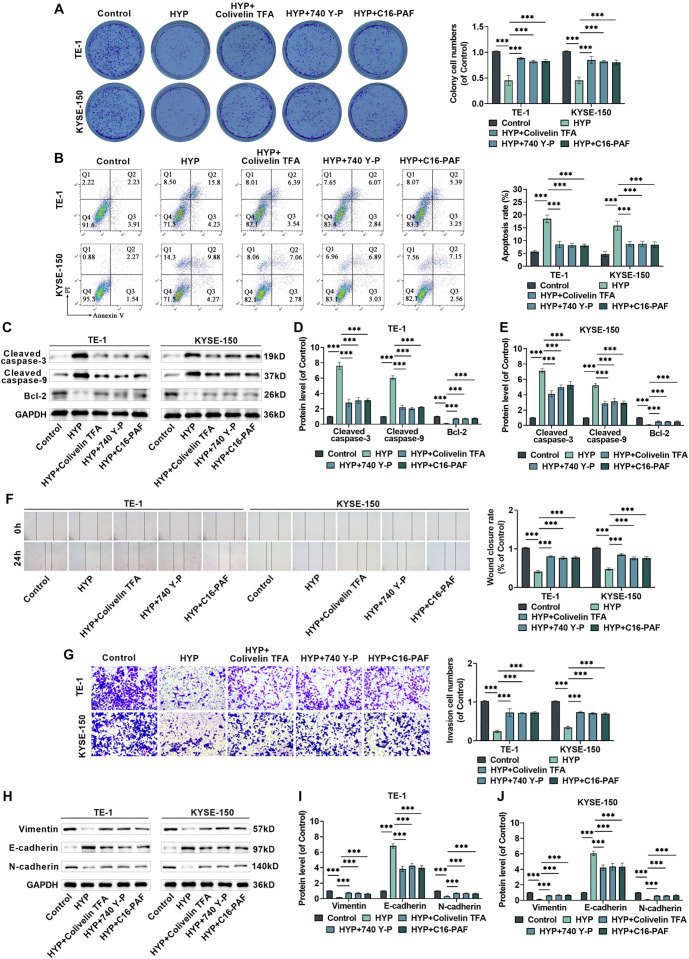
**HYP modulates the evolution of EC cells via the STAT3/AKT/ERK pathway.** (A) Measuring the cloning efficiency of TE-1 and KYSE-150 cells after being treated with HYP or STAT3/AKT/ERK signaling pathway agonists; (B) Assessing the impact of treatment with HYP or STAT3/AKT/ERK signaling pathway agonists on cell apoptosis rate; (C–E) Measuring the levels of Cleaved-Caspase3, Cleaved-Caspase9, and Bcl-2 proteins; (F) It is to observe the migration of EC cells 24 h post-scratching and quantify the wound closure rate, 10×, bar ═ 200 µm; (G) Evaluating the invasion of EC cells after being treated with HYP or STAT3/AKT/ERK signaling pathway agonists and to quantitatively analyze the cell invasion rate, 40×, bar ═ 50 µm; (H–J) Measuring the expression of Vimentin, E-cadherin, and N-cadherin proteins. *n* ═ 3. ***P < 0.001 vs corresponding control. HYP: Hyperoside; EC: Esophageal cancer; ERK: Extracellular signal-regulated kinase; STAT3: Signal transducer and activator of transcription 3; AKT: AKT serine–threonine kinase.

The effects of the STAT3 agonist (Colivelin TFA), AKT agonist (740 Y-P), and ERK agonist (C16-PAF) on the anticancer activity of HYP were explored to determine whether HYP exerts its effects via the STAT3/AKT/ERK signaling pathway. HYP treatment impaired the clone formation ability of TE-l and KYSE-150 cells, but this was reversed by the addition of signaling pathway agonists (Figure 4A) FCM and western blot assays revealed that HYP promoted apoptosis in TE-l and KYSE-150 cells, an effect mitigated by the signaling pathway agonists (Figure 4B–4E). Furthermore, HYP inhibited migration, invasion, and EMT in these cells; however, these inhibitory effects were weakened upon the addition of agonists (Figure 4F–4J). These findings confirm that HYP suppresses EC cell proliferation, migration, invasion, and EMT, while promoting apoptosis, through the STAT3/AKT/ERK signaling pathway.

**Figure 5.**
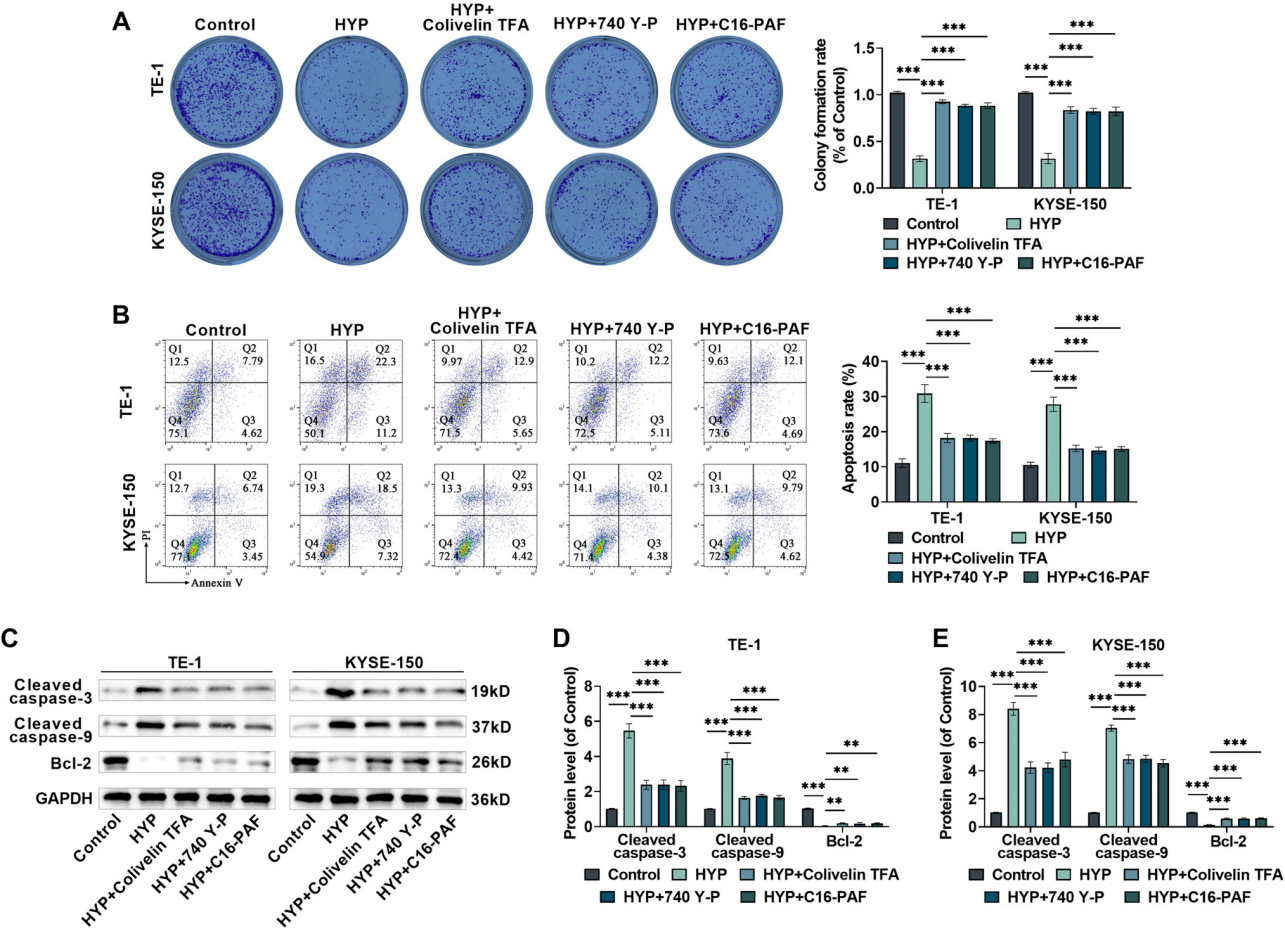
**HYP modulates the radiosensitivity of EC cells through the STAT3/AKT/ERK pathway.** (A) Following exposure to 6 MV X-ray radiation (4 Gy), clone formation experiments were conducted to test the cloning efficiency of cells treated with HYP or STAT3/AKT/ERK signaling pathway agonists; (B) Detecting cell apoptosis rate; (C–E) Evaluating the expression levels of Cleaved-Caspase3, Cleaved-Caspase9, and Bcl-2 proteins. *n* ═ 3. **P < 0.01, ***P < 0.001 vs corresponding control. (The control group underwent X-ray radiation, while the other groups were treated with HYP and signaling pathway agonist post-X-ray radiation.) HYP: Hyperoside; EC: Esophageal cancer; ERK: Extracellular signal-regulated kinase; STAT3: Signal transducer and activator of transcription 3; AKT: AKT serine–threonine kinase.

### HYP regulates EC cell radiosensitivity through the STAT3/AKT/ERK signaling pathway

HYP regulates EC cell radiosensitivity through the STAT3/AKT/ERK signaling pathway. To investigate whether HYP enhances the radiosensitivity of EC cells via the STAT3/AKT/ERK signaling pathway, we conducted a series of experiments. Results from the clonogenic assay demonstrated that HYP increased the inhibitory effect of X-rays on EC cell clone formation. However, this enhancement was attenuated when signaling pathway agonists, such as Colivelin TFA, 740 Y-P, or C16-PAF, were introduced (Figure 5A). Additionally, HYP amplified the pro-apoptotic effects of X-rays on EC cells (Figure 5B) and increased the expression levels of Cleaved-Caspase3 and Cleaved-Caspase9, while reducing Bcl-2 expression (Figure 5C–5E). In contrast, these effects were mitigated in the presence of the signaling pathway agonists. These findings suggest that HYP may improve the radiosensitivity of EC cells by acting through the STAT3/AKT/ERK signaling pathway.

### HYP suppresses the malignant progression of EC and enhances radiosensitivity by modulating the STAT3/AKT/ERK pathway

**Figure 6.**
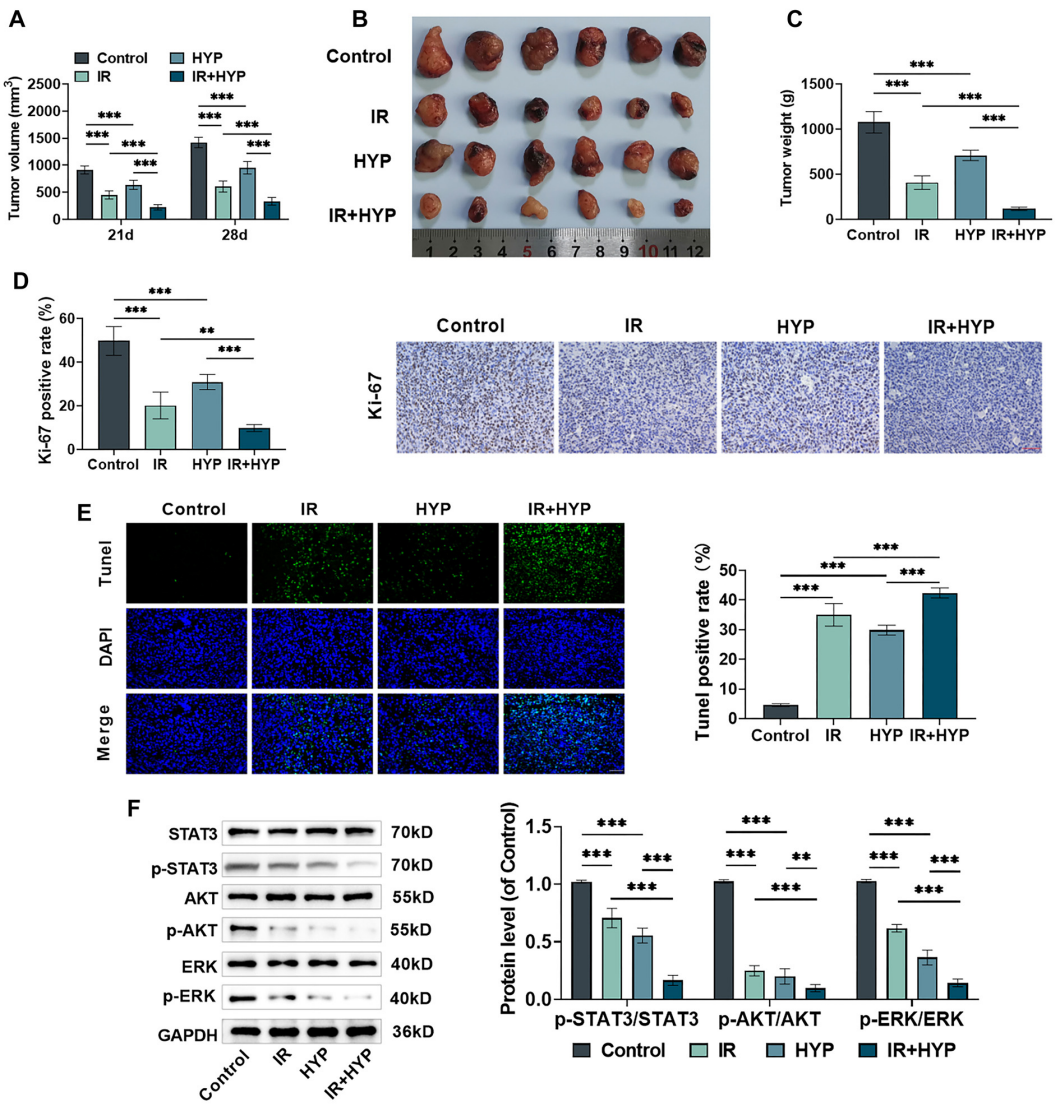
**HYP affects the evolution and radiosensitivity of EC cells by regulating the STAT3/AKT/ERK pathway.** On the 21st and 28th days after establishing subcutaneously-grafted tumor models, the sizes of subcutaneous tumors were measured using a caliper to calculate the tumor volumes (A). On the 28th day, the mice were put to death, the tumor was photographed (B), and the tumor mass was weighed (C). (D) Immunohistochemistry was used to detect Ki-67 protein expression in the tumor tissues, with brown–yellow stained cells indicating Ki-67-positive cells, 40×, bar ═ 50 µm. (E) TUNEL assay was conducted to evaluate the extent of apoptosis within the tumor tissue samples, with green fluorescence indicating TUNEL-positive cells, 40×, bar ═ 50 µm. (F) Western blot analysis was conducted to determine the expression of STAT3/AKT/ERK pathway proteins in the tumor tissues. *n* ═ 6. **P < 0.01, ***P < 0.001 vs corresponding control. (The control group is the blank control; the IR group underwent radiation treatment; the HYP group received oral HYP treatment; and the IR+HYP group received radiation treatment and oral HYP treatment.) HYP: Hyperoside; EC: Esophageal cancer; ERK: Extracellular signal-regulated kinase; STAT3: Signal transducer and activator of transcription 3; AKT: AKT serine–threonine kinase.

To investigate the effects of HYP on tumor growth *in vivo*, we established a subcutaneous transplantation tumor model in nude mice. Both HYP and X-ray treatments significantly inhibited tumor growth, as evidenced by reductions in tumor volume and mass (Figure 6A–6C). Notably, this inhibitory effect was further amplified when HYP was combined with X-ray treatment. Immunohistochemical analysis revealed that treatment with either HYP or X-ray alone decreased the Ki-67 positivity rate in tumor tissues (a marker for proliferation; Figure 6D) and increased the TUNEL positivity rate (a marker for apoptosis; Figure 6E). These effects were even more pronounced with the combined HYP and X-ray treatment.

Furthermore, both HYP and X-ray markedly reduced the phosphorylation levels of ERK, AKT, and STAT3 proteins in tumor tissues (Figure 6F). The combination treatment exhibited a more substantial inhibitory effect on these signaling pathways compared to either treatment alone. These findings suggest that HYP suppresses the malignant progression of EC and enhances the radiosensitivity of EC cells by modulating the STAT3/AKT/ERK pathway.

## Discussion

With its insidious onset, aggressive invasiveness, and lack of precise treatment options, esophageal cancer (EC) remains one of the leading global causes of cancer-related deaths [30, 31]. HYP, known for its diverse biological activities, holds significant potential for broad therapeutic applications in treating various diseases, including sepsis, arthritis, myocardial infarction, pulmonary fibrosis, and cancer [32–34]. Recent research has increasingly focused on the role of HYP in suppressing tumor progression. For instance, Hu et al. [35] demonstrated that HYP promotes the expression of forkhead box protein O1, thereby reducing the viability of non-small-cell lung cancer cells, inducing apoptosis, and inhibiting cell propagation in both *in vitro* and *in vivo* models. Similarly, our study revealed that HYP significantly reduced the viability of EC cells, inhibited their proliferation, migration, and invasion, and promoted apoptosis. Epithelial-to-mesenchymal transition (EMT) is a process through which epithelial cells acquire mesenchymal traits, contributing to embryogenesis, organ development, tissue formation, and tumor metastasis and invasion [36]. EMT is typically characterized by decreased expression of E-cadherin and increased levels of mesenchymal markers, such as N-cadherin, Vimentin, and Snail proteins [37]. In this study, HYP was found to upregulate E-cadherin expression while downregulating Vimentin and N-cadherin, indicating that HYP can inhibit EMT in EC cells. Furthermore, the Bcl-2 protein family, known for suppressing mitochondria-mediated apoptosis [38], exhibited reduced expression in EC cells following HYP treatment. This suggests that HYP may promote apoptosis in EC cells via intrinsic apoptotic pathways. RT is a key treatment modality for EC, particularly for patients with locally advanced or unresectable tumors, or those unsuitable for or unwilling to undergo surgery [39]. Radiation therapy is known to induce the production of heat shock proteins, tumor-associated antigens, and calreticulin, which promote dendritic cell maturation, enhance tumor tissue penetration, and trigger immune responses against cancer [40, 41]. However, RT resistance remains a major challenge, leading to local recurrence of EC. Our findings showed that HYP enhanced the inhibitory effects of X-rays on EC cell proliferation and amplified X-ray-induced apoptosis, indicating that HYP improves the radiosensitivity of EC cells. Additionally, *in vivo* experiments demonstrated that while both HYP and X-ray treatments individually suppressed tumor progression, their combined use exhibited a synergistic effect, further confirming HYP’s role in enhancing EC radiosensitivity. The progression of cancer involves complex mechanisms regulated by multiple signaling pathways. Among these, the STAT3, Akt, and ERK pathways play critical roles in cancer cell proliferation, survival, and angiogenesis and are often associated with poor prognosis and drug resistance [42–44]. For instance, Safi et al. [45] found that quercetin decreased pERK1/2, pAKT, and pSTAT3 protein levels in breast cancer cells, enhancing their sensitivity to docetaxel. In our study, HYP significantly reduced the phosphorylation of ERK, STAT3, and AKT proteins in EC cells. However, the addition of signaling pathway agonists partially reversed the inhibitory effects of HYP on EC progression. This suggests that HYP may exert its anticancer effects by suppressing the ERK/STAT3/AKT signaling pathway. Notably, the addition of a STAT3 agonist increased the phosphorylation levels of STAT3, AKT, and ERK, while AKT or ERK agonists only elevated the phosphorylation of AKT and ERK, without affecting pSTAT3 levels. This indicates that STAT3 activation occurs upstream of ERK and AKT. Consistent with these findings, Saxena et al. [46] reported that inhibiting the JAK/STAT pathway significantly reduced AKT and ERK phosphorylation, highlighting JAK/STAT’s upstream regulatory role. Furthermore, both *in vitro* and *in vivo* experiments demonstrated that HYP combined with X-ray treatment further reduced phosphorylation levels of ERK, STAT3, and AKT proteins compared to X-ray treatment alone. This finding suggests that HYP enhances radiosensitivity in EC cells, potentially by targeting the ERK/STAT3/AKT signaling pathway.

## Conclusion

Our findings demonstrated that HYP attenuates the viability of EC cells, inhibits their propagation, migration, invasion, and EMT, and enhances their sensitivity to X-ray treatment. Furthermore, HYP suppressed tumor growth in mice. Importantly, we identified a novel mechanism by which HYP disrupts the STAT3/AKT/ERK signaling pathway, impairing EC malignant progression both *in vitro* and *in vivo*, while improving the radiosensitivity of EC cells. In summary, HYP prevents EC malignant progression and enhances the efficacy of RT, underscoring its potential for clinical treatment of EC and the need for further investigation. However, the limitations of animal models, which do not fully replicate human metabolism and toxicity, necessitate extensive follow-up studies to confirm the safety and efficacy of HYP. Future research should also examine the effects of varying HYP concentrations on EC cell migration, invasion, and apoptosis to provide more comprehensive insights.

## Supplemental data

**Highlights:**

1. HYP prevented EC cells from invasion, proliferation, migration, and EMT, and induced apoptosis.
2. HYP heightened the radiosensitivity of EC cells.
3. HYP suppressed the malignant progression of EC and heightened radiosensitivity via the STAT3/AKT/ERK pathway.
4. HYP inhibited tumor growth in mice and enhanced X-ray’s inhibitory effect on tumor growth.

## Data Availability

The data that support the findings of this study are available from the corresponding author, FengLi Song, upon reasonable request.
